# Effect of Thyroxine and Thyrotropin on Bone Mineral Density in Postmenopausal Women: A Systematic Review

**DOI:** 10.7759/cureus.26353

**Published:** 2022-06-26

**Authors:** Prakar Poudel, Roopa Chalasani, Mastiyage R Goonathilake, Sara Waqar, Sheeba George, Wilford Jean-Baptiste, Amina Yusuf Ali, Bithaiah Inyang, Feeba Sam Koshy, Kitty George, Lubna Mohammed

**Affiliations:** 1 Research, California Institute of Behavioral Neurosciences & Psychology, Fairfield, USA

**Keywords:** postmenopausal women, bone mineral density, thyrotropin, thyroxine, dexa

## Abstract

The effect of thyroid hormones on bone mineral density in postmenopausal women has been reported but is not completely established. To better understand this relationship, this systematic review of the reported association, differences, and effects of thyroxine and/or thyrotropin levels on bone mineral density was conducted. An electronic literature search was conducted on MEDLINE, PMC, Google Scholar, Cochrane Library, and Science Direct from inception to April 2022; 20 studies were identified which include five quasi-experimental studies, three community cohort studies, and 12 hospital-based cross-sectional studies. Following an extensive evaluation, it was difficult to conclusively determine the association or effect of thyroxine or thyrotropin levels on bone mineral density in postmenopausal women. It is therefore suggested to conduct additional non-randomized or randomized control studies on this topic for the benefit of postmenopausal women. Particular attention should be given to the adjustment of age, body mass index, smoking status, alcohol consumption, and dietary calcium in future research on this topic for rigorous analysis.

## Introduction and background

Menopause is the mark of the end years of reproduction in a woman’s life when she naturally stops having menstruations. It happens when the ovaries stop making the estrogen hormone, which is responsible for her monthly menstrual period [1]. The National Institute of Aging has defined menopause as a time point at 12 months after a woman’s last period. The transitory period leading to this point of menopause is called peri-menopause. The natural average age of menopause is 51 years unless there is no pathological condition. However, it varies from ages 45 to 55 years. These variations may due to numerous lifestyle factors, exposure to hormones, genetics, race, ethnicity, and other factors [2].

A gradual loss of bone mass, usually after 50 years of age, is the natural phenomenon of the aging process. A general pattern of bone loss in aging, with the parameter of areal bone mineral density (BMD) assessed by dual-energy x-ray absorptiometry (DXA), had been well documented over many years [3]. Areal BMD is expressed in grams of calcium per square centimeter (g/cm^2^), which can also be plotted in terms of standard deviations of T and Z scores. T score denotes the standard deviation (SD) of measurement compared with the healthy young (age 20-40 years) population, whereas the Z score denotes the standard deviation of measurement compared with the population adjusted for age, weight, and ethnic group. According to the WHO criteria, a T-score < - 2.5 SD was defined as osteoporosis, between -2.5 and -1.0 SD as osteopenia, and > -1.0 SD as normal [4].

Women lose BMD more rapidly than men, especially with the onset of menopause. The loss is more noticeable in trabecular bone rather than in the cortical bone of the lumbar spine (LS), femoral neck(FN), pelvis/ total hip(TH), and ultra distal wrist [3]. During the first eight years of postmenopausal time frame, the accelerated bone loss is due to bone resorption being favored against the bone formation process [5]. The significant increase in bone mass of postmenopausal women after estrogen hormone administration depicts that estrogen is a regulator of such bone remodeling [6].

**Figure 1:**
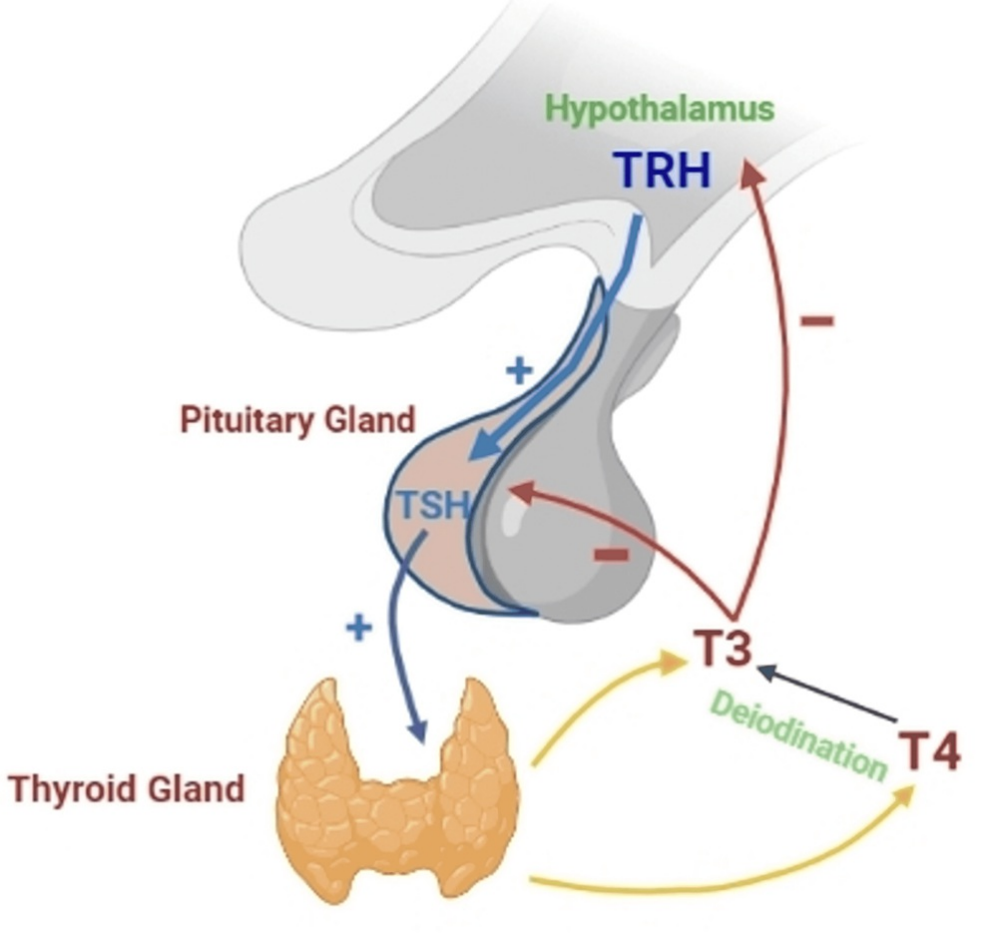
Hypothalamic-pituitary-thyroid axis. TRH: thyrotropin-releasing hormone; TSH: thyroid-stimulating hormone; T4: thyroxine; T3: triiodothyronine; (+): stimulatory effect; (­–): inhibitory effect. Figure created by the first author with BioRender.com.

Another regulatory mechanism also exists in the hypothalamic-pituitary-thyroid axis as illustrated below in Figure 1. The stimulatory effect of thyrotrophin/thyroid-stimulating hormone (TSH) on the synthesis and release of thyroid hormones (thyroxine/T4 and triiodothyronine/T3) from the thyroid gland is regulated by the counter-inhibitory effect of T3 and T4 on the pituitary gland to release TSH. Therefore, in regard to primary thyroid disorders that are more common, TSH level has a logarithmic inverse relationship to free T4 (fT4) and T3 level [7].

In addition, thyroid hormones and TSH levels are also altered with increasing age in postmenopausal women [8]. An extremely high level of thyroid hormones is a well-recognized risk factor for low BMD [9,10]. However, the effects of subclinical hyperthyroidism or hypothyroidism on BMD remain unclear. Furthermore, the influences of T4 replacement and suppressive therapy on BMD remain questionable. The association of TSH level with BMD had also been reported [11-13], along with discrepant results from some researchers [14,15].

The lifetime risk of hip and vertebral fractures in postmenopausal women is high with low BMD [4]. With such risk, the conflicting data on the bony effect of thyroid hormones or TSH brings challenges during titration of levothyroxine dose in replacement or suppressive therapy and also monitoring the bone status of the patients on hormone replacement therapy. There is no consensus demonstrating the effect of T4 levels of either endogenous or exogenous sources, or TSH levels on BMD in postmenopausal women. Therefore, this systematic review was conducted to examine the evidence on the correlation or effect of T4 level and/or TSH level on BMD in postmenopausal women.

## Review

Methods

This systematic review was carried out using the Preferred Reporting Items for Systematic Reviews and Meta-Analyses (PRISMA) 2020 guidelines.

Eligibility Criteria

The studies were selected based on the participants, exposure or intervention, and outcomes or effect (PIO) elements with or without a comparison group (C). Participants were postmenopausal women, exposure or intervention with thyroxine/TSH level, comparison with euthyroid women or within the subgroups, and effect measured in BMD. Table 1 and Table 2 enlist inclusion criteria and exclusion criteria respectively.

**Table 1:** Inclusion criteria checklist. DOI: digital object identifier; TSH: thyroid-stimulating hormone; BMD: bone mineral density.

Inclusion criteria
Articles with available DOI number published in English language or any other languages that could be retrieved using automatic Google translate
Reports with postmenopausal female participants with a mean or median age of 45 years and above
Reports with available measured levels of exogenous or endogenous thyroid hormones or TSH irrespective of treatment indications or disease pathology of study participants
Reports with available BMD data of lumbar spine or femoral neck or hip bone or lower arm irrespective of whether this is the endpoint or not

**Table 2:** Exclusion criteria checklist. SERM: selective estrogen receptor modulator; CKD: chronic kidney disease; ESRD: end-stage renal disease.

Exclusion criteria
Case report, case series, editorials, opinions, views and commentaries, literature review, conference proceedings, poster presentations, and any report with incomplete peer review.
Reports with study participants using oral contraceptives, menopausal hormonal therapy, and medications that might affect bone metabolism (calcium, Vitamin D or analogues, bisphosphonates, SERMs, diuretics, lithium, corticosteroids, antidepressants, or antipsychotics)
Reports with study participants having CKD, ESRD, ileal resection, rheumatic bone diseases

Search Strategy and Databases

The search was performed systematically with the use of keywords, Medical Subject Headings (Mesh) search blocks with BOOLEAN operators, wherever required, along with available filters using PubMed, PubMed Central (PMC), Cochrane Library, Google Scholar, and Science Direct databases. The last date of the search for all databases was April 3, 2022. The search strategy used in the process is listed in Table 3.

**Table 3:** Search strategy in databases with results.

Databases	Keywords	Search strategy	Filters	Search result
PubMed	Thyroxine, Thyroid Hormones, Levothyroxine, Thyrotrophin, TSH, Thyroid stimulating hormone, Bone Density, Bone mineral density, Bone mineral content, DEXA scan, DXA scan, Post menopause, Menopause	#1 Thyroxine OR “Thyroid Hormones” OR Levothyroxine OR Thyrotrophin OR TSH OR “Thyroid stimulating hormone” OR (( "Thyroxine/administration and dosage"[Majr] OR "Thyroxine/adverse effects"[Majr] OR "Thyroxine/blood"[Majr] OR "Thyroxine/drug effects"[Majr] OR "Thyroxine/therapeutic use"[Majr] OR "Thyroxine/therapy"[Majr] OR "Thyroxine/toxicity"[Majr] )) OR ( "Thyrotropin/administration and dosage"[Majr] OR "Thyrotropin/adverse effects"[Majr] OR "Thyrotropin/blood"[Majr] OR "Thyrotropin/drug effects"[Majr] OR "Thyrotropin/therapeutic use"[Majr] OR "Thyrotropin/therapy"[Majr] OR "Thyrotropin/toxicity"[Majr] ) #2 “Bone Density” OR “Bone mineral density” OR “Bone mineral content” OR “DEXA scan” OR “DXA scan” OR ( "Bone Density/drug effects"[Majr] OR "Bone Density/etiology"[Majr] ) #3 “Post menopause” OR Menopause OR (( "Postmenopause/blood"[Mesh] OR "Postmenopause/drug effects"[Mesh] OR "Postmenopause/etiology"[Mesh] OR "Postmenopause/metabolism"[Mesh] )) OR ( "Osteoporosis, Postmenopausal/blood"[Mesh] OR "Osteoporosis, Postmenopausal/drug therapy"[Mesh] OR "Osteoporosis, Postmenopausal/etiology"[Mesh] OR "Osteoporosis, Postmenopausal/metabolism"[Mesh] OR "Osteoporosis, Postmenopausal/prevention and control"[Mesh] OR "Osteoporosis, Postmenopausal/therapy"[Mesh] ); final search: #1 AND #2 AND #3	Female, Human, Middle Aged, Aged 45+ years	168
PMC	Not used	Selected 3 MeSH Major Topics: Thyroxine, Bone Density, Postmenopause in 3 rows with AND Boolean : ((Thyroxine[MeSH Major Topic]) AND Bone Density[MeSH Major Topic]) AND Postmenopause[MeSH Major Topic]	None	1
Google Scholar	“Thyroxine, TSH, thyrotropin, BMD, postmenopause” with Boolean	(thyroxine OR TSH OR thyrotropin) AND BMD AND postmenopause	None	12800 (First 415 records were identified)
Cochrane Library	Not used	#1 MeSH descriptor: [Thyroxine] explode all trees; #2 MeSH descriptor: [Bone Density] explode all trees; #3 ; MeSH descriptor: [Postmenopause] explode all trees; final search: #1 AND #2 AND #3	None	5
Science Direct	Thyroxine, TSH, thyrotropin, BMD, postmenopause	(thyroxine OR TSH OR thyrotropin) AND BMD AND postmenopause	Research article	25

Selection Process

**Figure 2:**
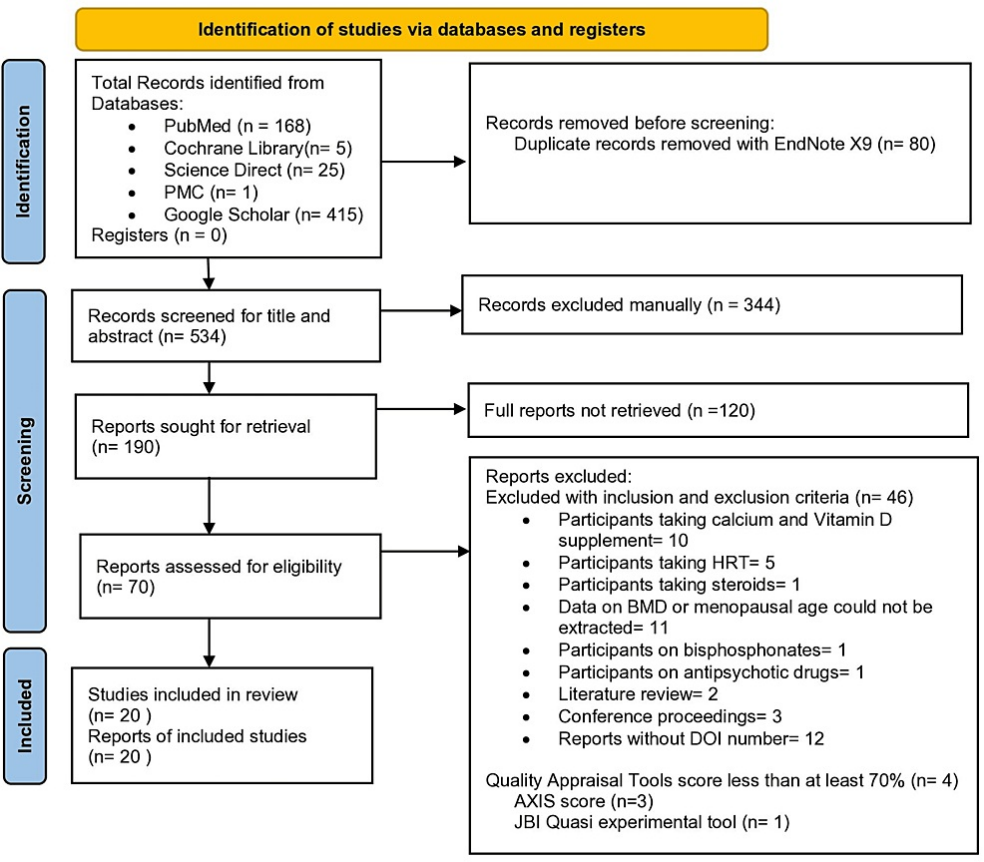
PRISMA flow chart of the review. PRISMA: preferred reporting items for systematic reviews and meta-analyses; HRT: hormone replacement therapy; BMD: bone mineral density; DOI: digital object identifier; AXIS: appraisal tool for cross-sectional studies; JBI: Joanna Briggs Institute.

There were a total of 614 records from all five databases using various search strategies across databases. 80 duplicate records were removed using EndNote X9 version and manually. The remaining 534 records were initially screened based on the titles and abstracts by the first author without any automation tool, excluding irrelevant studies. Hence, 190 reports were sought for retrieval of free full text; 70 reports were assessed for eligibility, and 46 articles did not fulfill the inclusion and exclusion criteria and were thus excluded. The complete PRISMA flow diagram of this review is shown in Figure 2.

Finally, a quality assessment for each article was done, and those studies with a score at or greater than 70% were accepted in the review. We used the following quality assessment tools: Cochrane Risk of Bias Tool for randomized control trial, JBI appraisal checklist for quasi-experimental study, Newcastle Ottawa scales for case-control and cohort study, and AXIS tool for cross-sectional study. The final studies to be included were decided by the first and second authors working independently for quality assessment of articles. The decision of the third author was sought in case of disagreement.

Data Synthesis

The outcome variables of interest were BMD or T score or Z score of the lumbar spine (LS), femoral neck (FN), total hip (TH), or trochanter. The key information of the selected studies was organized on a data sheet with the name of the first author, publication year, country, study design, study settings, study participants, comparison groups, thyroid disorder or other comorbidities, fT4 reference level, TSH reference level, the outcome measure of interest, statistical analysis and conclusion. All the relevant data from the included studies were extracted to an excel sheet form (Microsoft Corporation, 2010 version) by the first reviewer. No automated tool was used for the data extraction process.

Results

Quality Screening and Bias Risk

The screened 70 reports were first assessed using the inclusion and exclusion criteria. Then, the obtained screened reports were further assessed for their quality and risk of bias using appropriate tools which are well summarized in Tables 4-5. Only those reports with allotted scores at or greater than 70% were finally included in the review.

**Table 4:** Assessment of quasi-experimental studies with JBI questionnaire tool of Figure 4. JBI- Joanna Briggs Institute. Y- Yes, N- No, UC- Unclear; Scores allotted: Y= 2, N= 0, UC= 1.

Studies	Question number									Total allotted scores
	1	2	3	4	5	6	7	8	9	Percentage calculated
Kung AW et al. [34]	Y	Y	UC	Y	Y	Y	Y	Y	Y	94.40%
La Vignera S et al. [35]	Y	Y	Y	Y	Y	Y	Y	N	Y	88.80%
Chung CW et al. [36]	Y	UC	Y	Y	Y	Y	Y	Y	Y	94.40%
Zhang P et al. [37]	Y	Y	N	Y	Y	Y	Y	N	Y	77.80%
Yoshihara A et al. [38]	Y	Y	Y	N	Y	Y	Y	Y	Y	88.90%
Rosario PW et al. [39]	Y	Y	N	N	Y	Y	Y	Y	N	66.67%

**Table 5:** Assessment of cross-sectional studies with AXIS questionnaire tool of Figure 3. AXIS: appraisal tool for cross-sectional studies; Y: yes; N: No; UA: unsure/unanswered; scores allotted: Y= 2, N= 0, UA= 1. Note: For question number 13 and 19; N=2, Y= 0, UA=1.

Studies	Question number																					Total allotted scores
	1	2	3	4	5	6	7	8	9	10	11	12	13	14	15	16	17	18	19	20	Percentage calculated	
Polovina S et al. [16]	Y	Y	N	Y	UA	UA	N	Y	Y	Y	UA	Y	N	N	UA	Y	Y	Y	N	Y	75%	
Deniz HC et al. [17]	Y	Y	N	Y	Y	UA	N	Y	Y	Y	UA	Y	N	N	UA	Y	Y	N	N	Y	72.50%	
Vendrami C et al. [18]	Y	Y	Y	Y	Y	Y	Y	Y	Y	Y	Y	Y	UA	UA	Y	Y	Y	Y	Y	Y	90%	
Ding B et al. [19]	Y	Y	Y	Y	Y	Y	N	Y	Y	Y	Y	Y	UA	N	UA	Y	Y	Y	Y	Y	80.00%	
Moon JH et al. [20]	Y	Y	UA	Y	Y	Y	N	Y	Y	Y	Y	Y	Y	N	Y	Y	Y	Y	N	Y	82.50%	
Ali TM et al. [21]	Y	Y	N	Y	UA	UA	N	Y	Y	Y	Y	Y	Y	N	UA	Y	Y	Y	N	Y	72.50%	
Reverter JL et al. [22]	Y	Y	Y	Y	Y	N	Y	Y	Y	Y	Y	Y	Y	UA	Y	Y	Y	N	N	N	77.50%	
Hoeg A et al. [23]	Y	Y	Y	Y	Y	Y	N	Y	Y	Y	Y	Y	Y	N	N	Y	Y	Y	Y	Y	75%	
Murphy E et al. [24]	Y	Y	UA	Y	Y	Y	N	Y	Y	Y	Y	Y	Y	N	N	Y	Y	Y	Y	Y	72.50%	
Chen CY et al. [25]	Y	Y	UA	Y	Y	UA	N	Y	Y	Y	Y	Y	Y	UA	UA	Y	Y	Y	N	Y	77.50%	
Park SH et al. [26]	Y	Y	UA	Y	Y	UA	N	Y	Y	Y	Y	Y	UA	N	UA	Y	Y	Y	N	N	75.00%	
Liu C et al. [27]	Y	Y	UA	Y	Y	N	N	Y	Y	Y	Y	Y	Y	UA	UA	Y	Y	Y	Y	Y	72.50%	
Toivonen J et al. [28]	Y	Y	Y	Y	Y	N	UA	Y	Y	Y	Y	Y	Y	N	Y	Y	Y	N	Y	Y	70%	
Rosario PW et al. [29]	Y	Y	Y	Y	Y	N	N	Y	Y	Y	Y	Y	Y	N	UA	Y	Y	Y	N	Y	72.50%	
De Mingo ML et al. [30]	Y	Y	N	Y	Y	N	Y	Y	Y	Y	Y	Y	N	N	Y	Y	Y	Y	Y	Y	80%	
Baldini M et al. [31]	Y	Y	N	Y	Y	UA	N	Y	Y	Y	Y	Y	Y	N	UA	Y	Y	N	UA	UA	65.00%	
Arnautovic HA et al. [32]	Y	Y	N	Y	Y	N	N	Y	Y	Y	Y	UA	Y	N	N	UA	UA	N	N	Y	57.50%	
Begić A et al. [33]	Y	Y	N	Y	N	UA	N	Y	Y	Y	Y	Y	Y	N	UA	Y	Y	N	N	Y	65.00%	

Characteristics of Reports

After quality screening and bias risk assessment, the final 20 studies and reports were included in the review. The relevant extracted information from those final included studies and reports are enlisted in Tables 6-8. The tables are categorized on the basis of study type and study setting.

**Table 6:** Community-based cross-sectional studies BMI: body mass index; fT3: free triiodothyronine; fT4: free thyroxine; TSH: thyroid-stimulating hormone; BMD: bone mineral density; LS: lumbar spine; FN: femoral neck.

Studies	Publication year	Country of study	Euthyroid study participants	Reference values			Outcome: BMD, T score, and Z score	Statistical analysis	Adjustment for analysis	Conclusion
				fT3(pmol/L)	fT4 (pmol/L)	TSH (mIU/L)				
Vendrami C et al. [18]	2021	Switzerland	533	Not available	12-22	0.27-4.20	LS, FN, Hip T-score	Spearman correlation and linear regression	Age, BMI, duration of Menopause	No correlation or association between fT4 and TSH with LS-BMD, FN BMD, hip BMD
Hoeg A et al. [23]	2012	Europe	1140	2.11-5.23	9.07-16.62	0.13-3.48	LS-BMD, Hip BMD	Stepwise regression	Age, BMI, smoking, Selenium	Association persist between higher fT4 level and hip BMD
Murphy E et al. [24]	2010	European countries	593	2.16-5.29	9.15-16.99	0.13-3.48	LS-BMD, Hip BMD	Stepwise regression	Age, BMI, smoking	Inverse correlation between hip BMD and fT4 level , No relation between TSH level and BMD level

**Table 7:** Hospital-based cross-sectional studies. DTC: differentiated thyroid cancer; OD: odds ratio; fT4: free thyroxine; TSH: thyroid-stimulating hormone; BMD: bone mineral density; LS: lumbar spine; FN: femoral neck; TH: total hip; ANOVA: analysis of variance.

Studies	Publication year	Country of study	Study participant	Comparison groups	Thyroid disorders and comorbidities	Reference level		Outcome of interest	Statistical analysis	Conclusion
						fT4 level (pmol/L)	TSH level (mIU/L)			
Liu C et al. [27]	2021	China	213	TSH level tertile subgroups in euthyroid range	Type 2 Diabetes Mellitus	Not available	0.55-4.78	LS, FN, hip joint BMD	Mann-Whitney U test, Kruskal Wallis test, multivariate regression analysis	Significant difference in FN and hip joint BMD of low TSH compared with high TSH, Positive association between BMD of FN and hip joint and TSH
Ali TM et al. [21]	2020	Iraq	100	Hyperthyroid, hypothyroid, euthyroid	Unavailable	9-20	0.25-5	LS-BMD, Left femur BMD	Binary Logistic Regression	Hyperthyroidism was the significant risk factor that causes osteoporosis with an OD ratio of 2.89 (1.15-7.24)
De Mingo ML et al. [30]	2018	Spain	84	Baseline self-control	Thyroidectomy for DTC with TSH suppression	Not available	Not applicable	LS-BMD	Multivariable Linear regression	Serum TSH level and duration of TSH suppression are not a predictive variable of LS-BMD
Chen CY et al. [25]	2018	Taiwan	Levothyroxine treatment group=32	Baseline self-control	Nonfunctioning benign nodular thyroid goiter	6.1-16.1	0.35-5.5	LS-BMD, FN-BMD, TH-BMD	Paired t-test	After two years, no significant difference observed in BMD of the treatment group
Ding B et al. [19]	2016	China	1097	Normal BMD, osteopenia, osteoporosis	Unavailable	7.8-15.7	0.5-5	Not applicable	ANOVA, multiple logistic, and linear regression	Significant differences in TSH levels among the 3 groups
Moon JH et al. [20]	2016	South Korea	273	None	Thyroidectomy for DTC with TSH suppression	11.4-23	0.3-4	LS BMD	Pearson correlation	No correlation between fT4 and TSH with LS-BMD
Rosario PW et al. [29]	2016	Brazil	Study group= 90, comparison group = 90	Subclinical hyperthyroid, euthyroid	Autonomous nodular thyroid disease	10.3-23	0.4-4	LS, FN-BMD	Fisher's exact test, Mann-Whitney U test	No significant difference between the two groups
Polovina S et al. [16]	2015	Serbia	Study group=27, comparison group= 51	Subclinical hyperthyroid, euthyroid	Autoimmune thyroiditis, toxic goiter	9-19.1	0.35-4.94	LS and FN-BMD, T-score	t-test/Mann-Whitney U test, Linear regression	Significant association between TSH, fT4, and FN BMD
Deniz HC et al. [17]	2014	Turkey	261	Hyperthyroid, hypothyroid, autoimmune thyroiditis, euthyroid	Hyperthyroidism, hypothyroidism, Autoimmune thyroiditis	11.5-21.8	0.27-4.2	LS, FN-BMD, T-score	ANOVA	No significant difference in BMD observed among the groups
Park SH et al. [26]	2008	South Korea	Study group=38, comparison group = 49	TSH suppressive group(<0.35) and non-suppressive group	Thyroidectomy for DTC with TSH suppression	Not available	Not applicable	LS-BMD, FN-BMD, T score, Z score	Unpaired t-test	No significant difference observed between these two groups
Reverter JL et al. [22]	2005	Spain	88	Levothyroxine suppressive therapy, euthyroid	Thyroidectomy for DTC with TSH suppression	10.2-24.45	0.3-5.5	LS-BMD, FN-BMD, T-score, and Z-score	t-test, Chi-square test	No difference was observed in BMD, T, and Z score between the therapy group and the control
Toivonen J et al. [28]	1998	Finland	10 patients and 12 comparison	TSH suppressive group and euthyroid group	Thyroidectomy for DTC with TSH suppression	8-24	0.06-4.1	LS, FN, Ward's triangle BMD	Two-sided t-test	No significant difference between the two groups

**Table 8:** Hospital-based quasi-experimental studies. DTC: differentiated thyroid cancer; fT4: free thyroxine; fT3: free triiodothyronine; TSH: thyroid-stimulating hormone; BMD: bone mineral density; LS: lumbar spine; FN: femoral neck; DR: distal radius; ANOVA: analysis of variance.

Studies	Publication year	Country of study	Study participants	Thyroid disorders or other comorbidities	Comparison groups	Reference values		Follow –up time	Outcome of interest	Statistical analysis	Conclusion
						fT4 level	TSH level				
Chung CW et al. [36]	2021	South Korea	164	Thyroidectomy for DTC with TSH suppression	TSH suppressive groups based on level (mean <1 mIU/L and >1 mIU/L)	11.45-23	0.3-4	2 years and 4 years	LS-BMD	t-test	No significant difference was observed between these two groups at 2 and 4 years of suppression
Zhang P et al. [37]	2018	China	225	Thyroidectomy for DTC with TSH suppression	TSH suppression groups based on level (mean <0.3 mIU/L and >0.3 as control), Baseline self-control group	6.6-24.8	0.3-4.6	6, 12, 24 months	LS-BMD	Paired and unpaired t-test, Pearson analysis	No significant difference between two different TSH level groups, no difference after treatment in 6, 12, 24 months compared with baseline in both TSH levels groups, and no correlation between fT4, fT3, and BMD level
Yoshihara A et al. [38]	2016	Japan	42	Graves’ disease	Baseline pre and post treatment self-control group	10.3-20.6	0.2-4.5	6,12,18,24 months	LS-BMD, FN-BMD, DR-BMD, T-score, Z score	Wilcoxon analysis, ANOVA	Significant differences were observed in FN-BMD from pre-treatment baseline after 12 months of achieving euthyroid state, Significant increase in BMD-FN after 6 months of achieving euthyroid state
La Vignera S et al. [35]	2012	Italy	110 treatment group, 50 control group	Normo-functioning nodular benign thyroid disease	Levothyroxine therapy and no therapy groups, Pre and post-exposure self-control group	Not available	0.49-4.67	2 years	LS-BMD	Paired and unpaired t-test	After two years, suppressive levothyroxine therapy decreases LS-BMD.
Kung AW et al. [34]	1995	China	15	Thyroidectomy for DTC with TSH suppression	Baseline pre and post-exposure self-control group (TSH maintained below <0.03 throughout the study)	Not available	Not applicable	2 years	LS-BMD, FN-BMD, Trochanter, Ward's triangle of Hip	Paired t-test	Significant bone loss at LS 5%, FN- 6.7%, trochanter-4.7%, Ward's triangle of hip-8.8%

Discussion

In this systematic review, we found that three community cohort studies were carried out among the population of European countries and concluded with mixed results. One of the cohort studies demonstrated that both fT4 and TSH levels within the euthyroid range are not associated with BMD of LS, FN, and hip [18]. However, another six-year cohort study demonstrated an inverse relationship between hip BMD and fT4 levels across the normal fT4 concentration range. This association was more evident in hip BMD at the beginning and after six years of follow-up. While the BMD of the lumbar spine was correlated with the fT4 level, only at the baseline of the study. The TSH had no relationship with the BMD [24]. Similarly, a large cohort study in the European population observed that a negative association persists between fT4 level and hip BMD [23]. These three cohort population studies have different reference ranges for fT4 and TSH, which could explain these discrepant results. Additionally, the adjustment for statistical analysis of alcohol consumption, duration of menopause, and smoking status was not present in every study.

Multiple hospital-based cross-sectional studies demonstrated that there was no significant difference in BMD level or T score or Z score between comparison groups with high, low, and normal fT4 and/or TSH levels [17,22,26,28-30]. Moreover, a study also found no significant correlation between fT4 level and TSH level with LS-BMD. There was also no difference in LS-BMD, in accordance with the duration of low TSH levels [20]. Also, another study with a baseline comparison of the TSH suppressive group showed no significant difference in BMD before and after two years of follow-up. However, the duration of TSH suppression was not adjusted in the study analysis [25].

In contrast, one study revealed a significant positive relationship between TSH level and the negative correlation between fT4 level and FN-BMD. This relationship was even evident after the adjustment of age, BMI and smoking [16]. Likewise, another study also found that hyperthyroidism was a significant risk factor for osteoporosis with the odds of developing osteoporosis being 2.89 with a high fT4 level. However, this study was small enough to have this conclusion [21]. Also, a study demonstrated a significant difference in both FN and hip BMD between low and high TSH level groups [27]. On the other hand, one study identified a significant difference in TSH levels between osteoporosis and normal groups. This study also predicted that the odds of having osteoporosis increase with low TSH levels. Further analysis even revealed that the women with osteoporosis had significantly higher fT4 levels and lower TSH levels in comparison with osteopenia or the normal group. However, this study was cross-sectional and the percentage of women did not reach a significant difference between subclinical hyperthyroidism and hypothyroidism, which can render these findings questionable [19]. Therefore, all of these studies signify a positive relationship between TSH level and the negative relationship of fT4 level with BMD value.

A total of five quasi-experimental studies were identified as a part of this systematic review. One of the studies concluded a significant decrement of 5%, 6.7%, and 4.7% in BMD at LS, FN, and trochanter site respectively, after keeping TSH levels below 0.03 mIU/L throughout two years of study. Although, the study participants were few [34]. Similarly, in another study, suppressive therapy that kept TSH level below 0.5 mIU/L for two years significantly reduced LS-BMD. But this finding was only apparent if BMI and smoking status were not adjusted [35]. A study with a different approach also found that treating hyperthyroidism can lead to a significant increase in FN-BMD after a minimum of six months of achieving euthyroid status. But, the duration of hyperthyroid status at the beginning of the study was not adjusted [38].

Inconsistent with earlier studies, two studies indicated an opposing conclusion, however, there are still gaps in these findings. One study found no significant difference in LS-BMD relative to TSH suppression groups (below 1 mIU/L and above 1 mIU/L). No difference was observed at either two years or four years of suppression. However, the researchers of this study did not adjust the analysis for BMI and smoking status [36]. Likewise, another study also depicted that there was neither a correlation between fT4 level and BMD level nor there was a difference in BMD before and after TSH suppression for as long as two years. However, this finding could have also been biased by the inclusion of patients with lung metastasis [37].

Limitations

This systematic review was done based on available studies. Even though the inclusion criteria include studies with mean or median age of more than 45 years, some cross-sectional studies have participants with an age range starting from 40 years [16]. The discrepancy is obvious with heterogeneity in terms of genetic variations, variation among reference range of thyroid hormones level, BMD value of population, and the variation in T and Z scores due to geographical, racial, and ethnical differences [24]. The assumption that smoking, alcohol consumption, and coffee use among study participants could be confounding factors that had not been fully adjusted in multiple studies. In addition, dietary calcium intake can be difficult to monitor and maybe a confounding factor in deviating BMD outcomes [22,34]. Neither the BMI, which is an obvious factor to influence BMD [17,20,22,25,26,28,29,35,36], nor the duration of exposure to T4 level or TSH level were adjusted in multiple studies [16,19]. This review also did not extract a portion of data from a report because of the discrepancy between the statement written in the result section and the information presented in Table number 2 [19].

## Conclusions

It was difficult to undoubtedly prove that T4 and TSH levels have an independent effect on BMD in postmenopausal women. Despite the demonstration of association with few studies, the overall conclusion was inconsistent and still remains questionable. Additional research must be conducted with a randomized or non-randomized controlled study design to be able to find the definitive effect. Moreover, the adjustment of age, BMI, smoking, alcohol consumption, duration of exposure, and dietary intake of calcium need to be made in studies for robust results. The clear conclusion on this topic can be beneficial for postmenopausal women while monitoring the bone status in thyroid disorders or during titration of medications that alters bone metabolism.

## Figures and Tables

**Figure 3:**
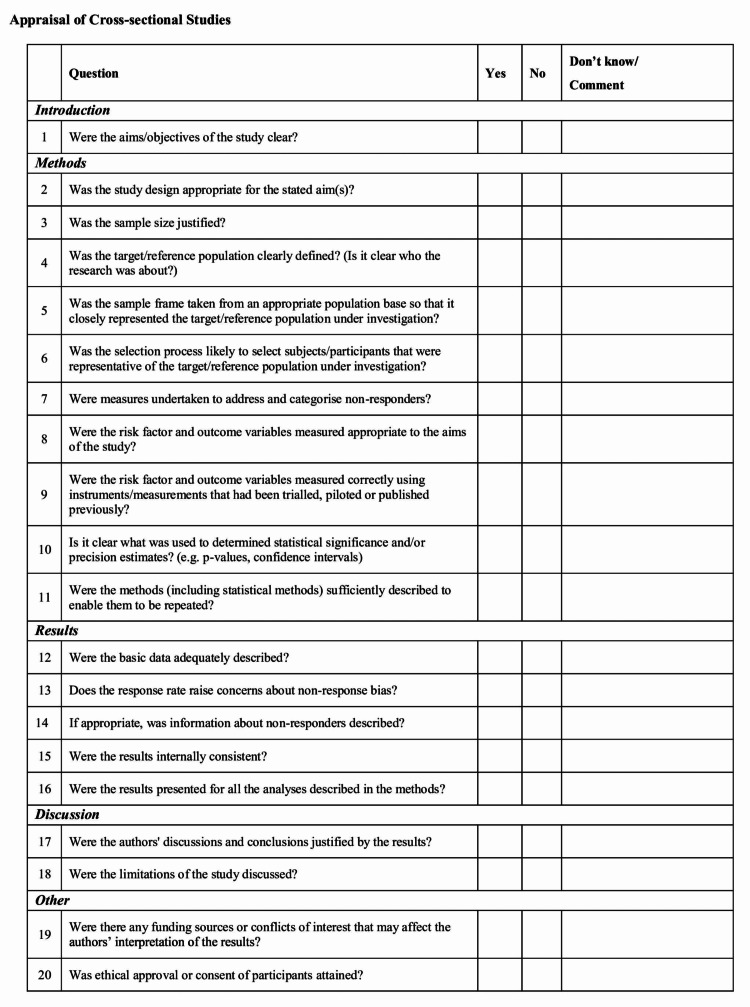
AXIS- Appraisal tool for cross-sectional studies.

**Figure 4:**
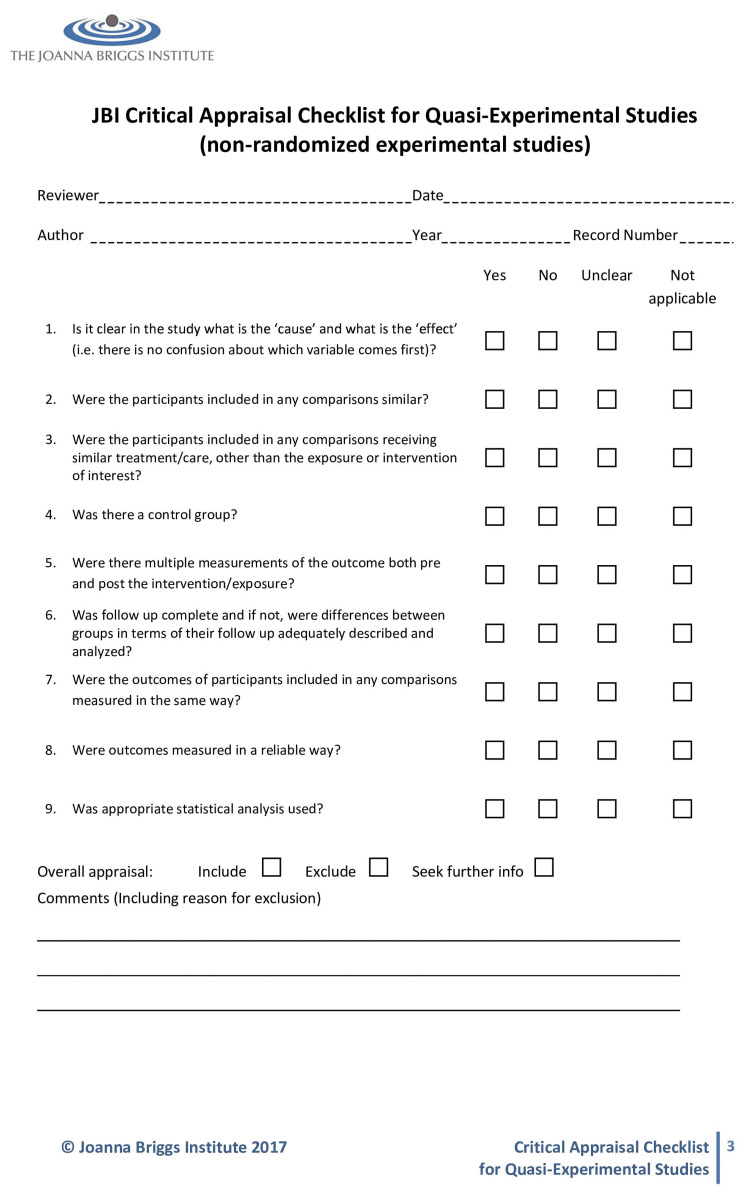
Joanna Briggs Institute (JBI) checklist for quasi-experimental studies.

## Appendices

 Appraisal of cross-sectional studies can be seen in Figure 3.

Figure 9 shows the Joanna Briggs Institute (JBI) checklist for quasi-experimental studies. 
